# Supplementary material for: Colour selective control of terahertz radiation using two-dimensional hybrid organic inorganic lead-trihalide perovskites
Source: Nat Commun. 2017 Nov 6;8:1328. doi: 10.1038/s41467-017-01517-0 (PMC5673065; doi:10.1038/s41467-017-01517-0)
Supplement: Supplementary file 1 — Supplementary Information [file 41467_2017_1517_MOESM1_ESM.pdf]

Supplementary Note 1: THz transmission through 2D perovskite/ Silicon bilayer structure

Two-dimensional hybrid perovskite/ silicon bilayer structures have been shown to exhibit enhanced absorption of THz upon photoexcitation. The samples were fabricated using the procedure described in the main text. Bilayer samples were excited from the perovskite side using tungsten-halogen lamp. Supplementary Figure 1 shows the THz transmission properties with increasing lamp flux for the n = 1 and n = 3 2D perovskite/ silicon samples. As with the n = 2 perovskite (Figure 1, main text), the n = 1 and n = 3 samples also show nearly 100% absorption of the incident THz radiation with increasing lamp intensity. When these materials are deposited on dielectric substrates, the perovskite films show minimal additional THz absorption upon photoexcitation.

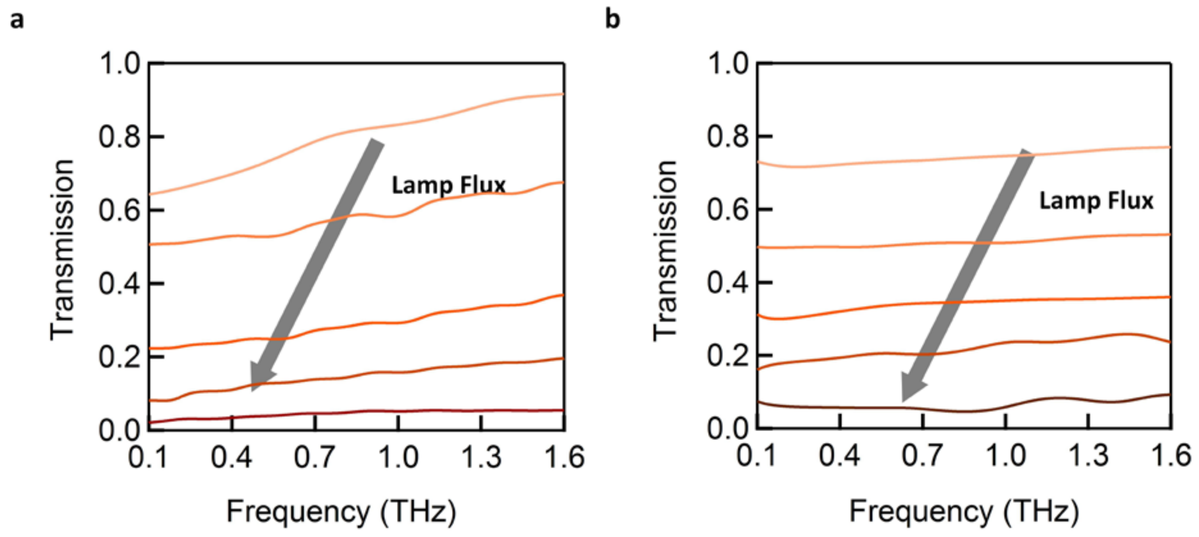

(a) (b)

Supplementary Figure 1 | THz transmission through photoexcited 2D perovskite/Si bilayers: **a** and **b,** THz transmission spectra through an n = 1 and n = 3 2D perovskite respectively, deposited on high resistivity Si wafers as a function of photoexcitation intensity. The direction of the arrow corresponds to increasing lamp flux from 0.1 W/cm^2 – 0.5 W/cm^2.

Supplementary Note 2: Photoinduced Free Carrier Absorption (FCA) in 2D perovskite/ Silicon bilayer structure using FTIR spectroscopy

Supplementary Figure 2 shows the photo-induced absorption (PA) characteristics of 2D perovskite/Si, 2D perovskite on a KBr (dielectric) substrate, and a bare Si substrate. The PA spectra measured in 2D perovskite/Si and bare Si samples both exhibit a $1/\omega^2$ characteristic of Drude FCA response; whereas the PA response of the 2D perovskite on dielectric did not follow those dynamics. This strongly suggests that the enhanced THz absorption in 2D perovskite/Si samples originates from free carriers in the Si substrate that are due to exciton dissociation in the Si substrate.

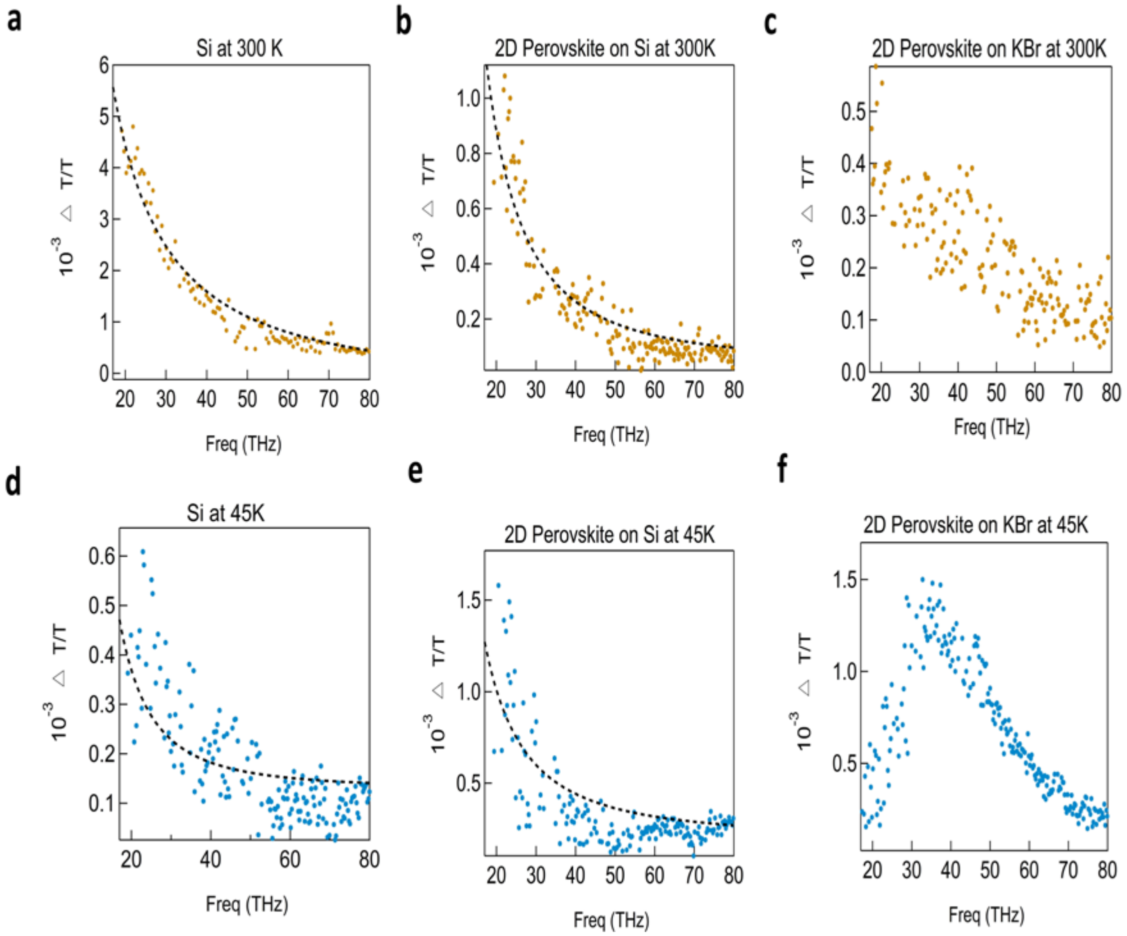

Supplementary Figure 2 | Photoinduced free-carrier absorption measured using FTIR : a – c Photoinduced absorption spectra for 2D perovskite/ Si, Si substrate and 2D perovskite on KBr measured at 300 K. **d – f** shows the corresponding spectra at 45 K.

Supplementary Note 3: Fabrication of 3D Perovskites

For preparing the 3D perovskite films, the $CH_3NH_3PbI_3$ precursor solution was made by mixing 0.23 g PbI_2, 0.42 g $PbCl_2$ and 0.80 g CH_3NH_3I together in anhydrous N,N-Dimethylformamide (DMF, 2.5 ml), and allowed to stir overnight on a hot plate at 60°C. A bright yellow solution was obtained, and cooled down to room temperature before use. Silicon substrates were cleaned and treated with O_2 plasma for 30 mins, so that the hydrophilic surface could allow for more uniform perovskite layers. The precursor solution was then spin coated at 3000 RPM for 30 seconds, and annealed at ~105°C for 90 mins. All processes were done in a glovebox filled with nitrogen (O_2/H_2O < 1 ppm).

Supplementary Note 4: Excitation Dependence of n=4 R-P and 3D perovskite

In contrast to the n = 1, 2, and 3 perovskite / silicon bilayer samples, the n = 4 sample did not exhibit obvious (optical) excitation wavelength specific THz absorption. This can be attributed to the low exciton binding energy and low energy edge states in n = 4 perovskite films associated with relaxed quantum confinement, when compared to films composed of lower n-value perovskites. The results are generally consistent with published data [1,2]. Supplementary Figure 3a shows excitation dependence for an n = 4 perovskite/ silicon bilayer sample. For comparison, the excitation dependent response of a 3D perovskite/ silicon structure is also shown in

Supplementary Figure 3b. In this case, the absorption is associated with free carriers generated in the perovskite films and does not allow for colour selective control of THz devices.

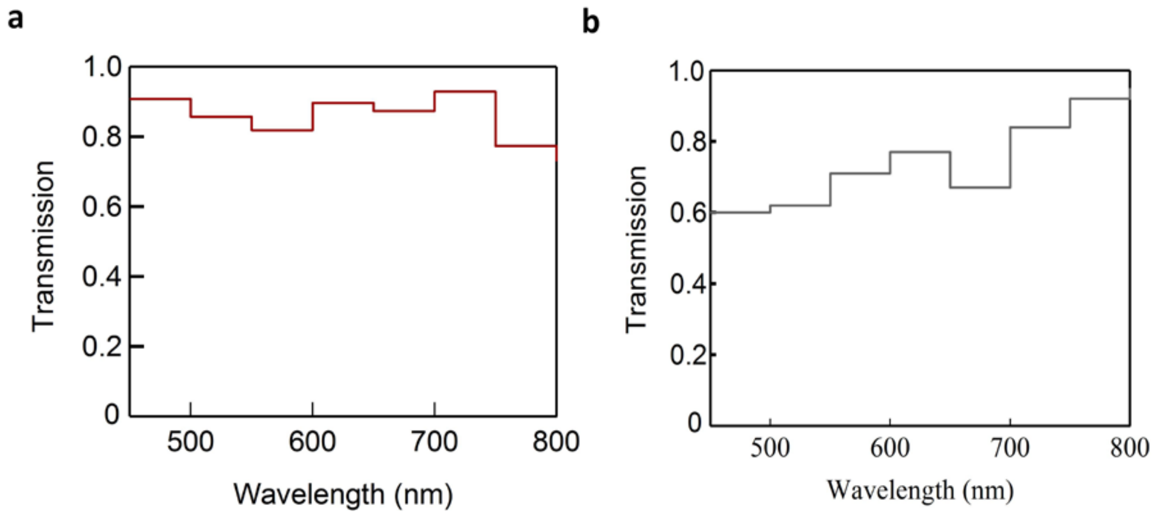

Supplementary Figure 3 | Excitation dependent spectra of the THz transmission: **a** and **b,** Wavelength selective THz transmission of the silicon/perovskite samples for the n = 4 layered (red) and the 3D (gray) perovskites respectively. In comparison to n = 1, 2, and 3 perovskites (Figure 2), the excitation dependent response does not allow for optical wavelength specific control of THz absorption.

Supplementary Note 5: Intensity modulation in excitation selective THz modulator

Supplementary Figure 4 shows the intensity dependent transmission spectra associated with the device shown in Figure 5a (main text). The device consists of 3 sets of resonances associated with three different aperture array sections fabricated on a high resistivity silicon substrate. By varying the intensity of the excitation source, the resonances can be suppressed in a controlled manner (i.e. as a function of the optical intensity). The dashed lines show intermediate levels (nearly 50 % and 25 % modulation levels), while the solid trace corresponds to nearly 100 %

attenuation of the associated resonance; thus, control of THz transmission is both excitation wavelength and intensity dependent.

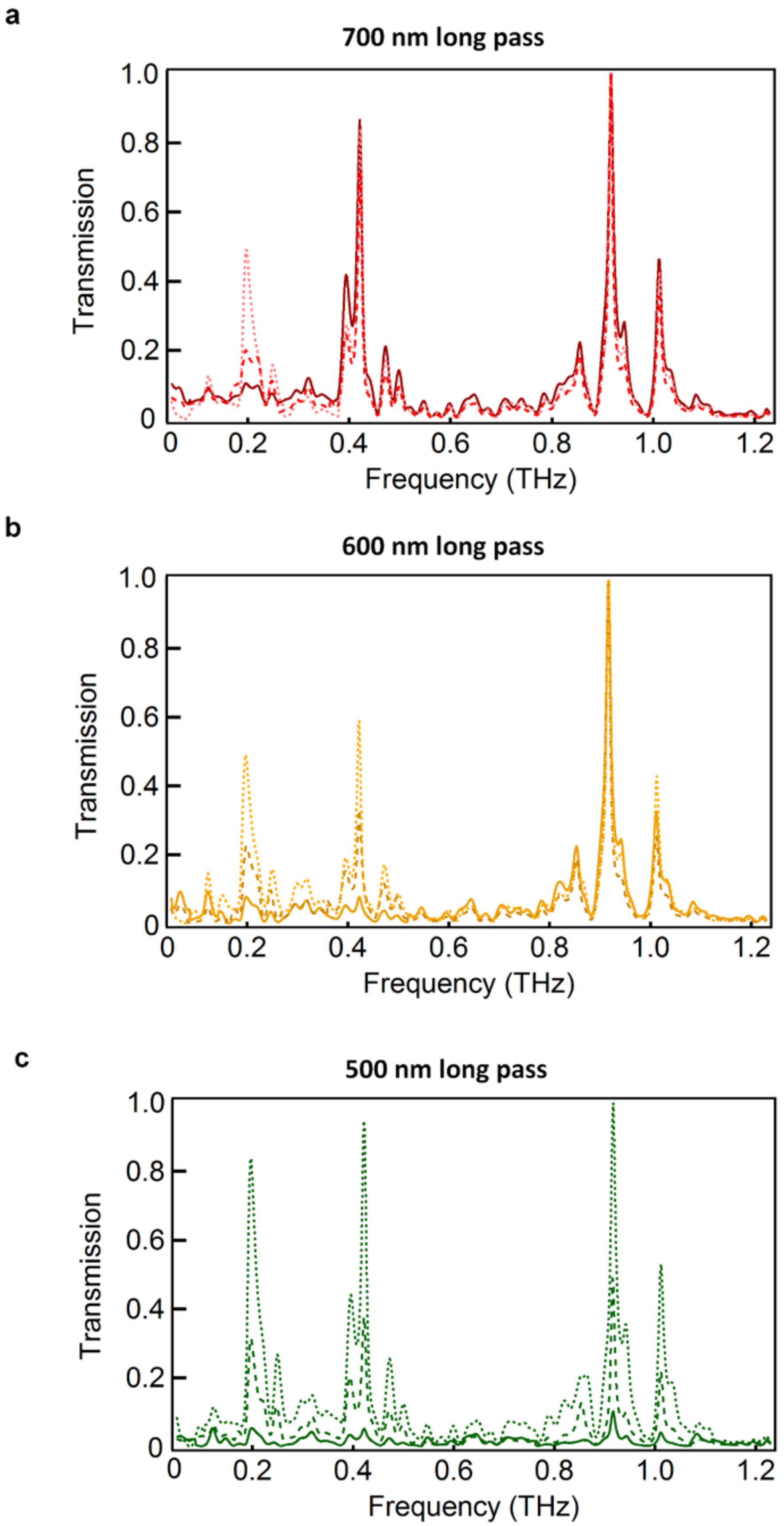
a
700 nm long pass
Transmission
Frequency (THz)
b
600 nm long pass
Transmission
Frequency (THz)
c
500 nm long pass
Transmission
Frequency (THz)

Supplementary Figure 4 | Intensity modulation in excitation selective THz modulator: **a** to **c,** Intensity modulation in selective resonances corresponding to 3 sections of colour selective THz plasmonic modulator as described in main text (Figure 5). Dashed to solid lines show increasing attenuation of THz resonance with increasing lamp flux.

Supplementary References:

[1] C. C. Stoumpos, *et al.,* Ruddlesden–Popper hybrid lead iodide perovskite 2D homologous semiconductors, *Chem. Mater.* **28**, 2852 (2016).

[2] J.-C. Blancon *et. al.,* Extremely efficient internal exciton dissociation through edge states in layered 2D perovskites, *Science* **355**, 1288 (2017).
